# Intradermal Testing With COVID-19 mRNA Vaccines Predicts Tolerance

**DOI:** 10.3389/falgy.2022.818049

**Published:** 2022-05-31

**Authors:** Florian Stehlin, Rima Mahdi-Aljedani, Loris Canton, Véronique Monzambani-Banderet, Alix Miauton, Cedric Girard, Kevin Kammermann, Sylvain Meylan, Camillo Ribi, Thomas Harr, Daniel Yerly, Yannick D. Muller

**Affiliations:** ^1^Division of Immunology and Allergy, University Hospital of Lausanne, Lausanne, Switzerland; ^2^Tropical, Travel and Vaccination Clinic, Center for Primary Care and Public Health (Unisanté), Lausanne, Switzerland; ^3^Division of Pharmacy, University Hospital of Lausanne, Lausanne, Switzerland; ^4^Adverse Drug Reactions - Analysis & Consulting (ADR-AC) GmbH, Bern, Switzerland; ^5^Infectious Diseases Service, University Hospital Lausanne and University of Lausanne, Lausanne, Switzerland; ^6^Division of Immunology and Allergy, University Hospital of Geneva, Geneva, Switzerland

**Keywords:** SARS-CoV-2, allergy, vaccine, polyethylene glycol, PEG, COVID-19, anaphylaxis, basophil activation test

## Abstract

**Background:**

The newly developed mRNA-based COVID-19 vaccines can provoke anaphylaxis, possibly induced by polyethylene glycol (PEG) contained in the vaccine. The management of persons with a history of PEG allergy or with a suspected allergic reaction after the first dose remains to be defined.

**Methods:**

In this real-life study, we defined two cohorts of individuals: one pre-vaccination including 187 individuals with high-risk profiles for developing anaphylaxis and a second post-vaccination including 87 individuals with suspected allergic reactions after the COVID-19 mRNA vaccine. Upon negative skin test with an mRNA vaccine, a two-step (10–90%) vaccination protocol was performed. Positive skin tests were confirmed with the basophil activation test (BAT).

**Results:**

Among 604,267 doses of vaccine, 87 suspected allergic reactions (5 after the booster) were reported to our division for further investigations: 18/87 (21%) were consistent with anaphylaxis, 78/87 (90%) were female, and 47/87 (54%) received the BNT162b2 mRNA vaccine. Vaccine skin tests were negative in 96% and 76% of the pre- and post-vaccination cohorts, respectively. A two-step vaccination was tolerated in 232/236 (98%) of individuals with negative tests. Four individuals experienced isolated asthmatic reactions during the two-step challenge. Vaccine-positive skin tests were consistently confirmed by BAT; CD63 and CD203c expression was selectively inhibited with ibrutinib, suggesting an IgE-dependent mechanism.

**Conclusion:**

Sensitization to SARS-CoV-2 mRNA vaccines can be detected with intradermal testing. Significantly more individuals were sensitized to mRNA vaccines in the post-vaccination cohort. A two-step 10–90%-vaccination protocol can be safely administered upon negative skin testing.

## Introduction

The newly developedanti-SARS-CoV-2 COVID-19 mRNA vaccines (BNT162b2 mRNA from Pfizer-BioNTech and mRNA-1273 from Moderna) represent a potential exit strategy from the ongoing COVID-19 pandemic but are associated with rare cases of anaphylaxis (1–3). The first severe cases of anaphylaxis were rapidly reported after the start of the worldwide vaccination campaign and led the US and European centers for disease prevention and control to recommend 15 min surveillance post-vaccination, which should be prolonged to 30 min in any patient with a history of severe anaphylaxis. The vaccination is currently contraindicated for persons with an allergic reaction to the COVID-19 vaccine or known allergies to any of its components. This includes polyethylene glycol (PEG), or macrogols, and polysorbate, the latter being known to cross-react with PEG, as it is constituted of PEG molecules (4).

In the US, the incidence of allergic reactions to mRNA vaccines was initially estimated to be 10 times higher than for other virus-based vaccines (2, 3). Based on a metanalysis regrouping 41,000,000 vaccinations and a more recent study, the incidence of SARS-CoV-2 vaccine anaphylaxis is estimated between 4.7 and 7.9 cases per million vaccinations (5–7). Expert opinions and a number of small case reports suggest that people with immediate allergic reactions to mRNA vaccines are likely sensitized to PEG (7–10). A large panel of international experts has recommended urgent research to define the utility of skin testing to identify the risk for allergic reactions to the vaccines or their excipients in allergic individuals (7).

For evident safety reasons, in our division, we rapidly established a routine diagnostic protocol consisting of vaccine drug skin testing and, if negative, this was followed by a two-step vaccination protocol (10–90%). This diagnostic protocol included two categories of individuals. The first one included a person before vaccination, with prior history of anaphylaxis to any injectable drug (intravenous, subcutaneous, or intramuscular) that contained PEG, polysorbate, or trometamol. Patients with a documented allergy to PEG and/or polysorbate were also included. In cases where the presence/absence of those excipients could not be clearly established, patients were also invited to get tested before vaccination. Thus, the centers for disease control and protection (CDC) recommended vaccination with precaution in these individuals (7). Patients with severe allergic reactions unrelated to injectable drugs (i.e., oral medication, pollen, food, Hymenoptera venom, pets, latex, and house dust) were vaccinated with 30 min surveillance. The second category included persons after vaccination that showed immediate-type allergic reactions following the first or second dose of vaccine.

Herein, we report the results of skin tests, basophil activation test (BAT), and clinical tolerability of a two-step vaccination protocol in patients with negative skin tests.

## Methods

### Study Approval

The study was approved by the local Ethics Committee of the canton of Vaud, Switzerland (BASEC number 2021-00735). Written informed consent was obtained from all participants.

### Pre-vaccination Cohort

The canton of Vaud in Switzerland has over 800,000 inhabitants. To get an appointment for the vaccination, every person went through a standardized evaluation, either online (https://coronavax.unisante.ch/evaluation) or via a hotline (Supplementary Figure 1). In the assessment, every person had to respond to the following questions:

1. Do you have allergies?
2. Do you have documented allergy to any of the vaccine components^*^ [^*^Polyethylene-Glycol (PEG, macrogol), polysorbate, tromethamine (TRIS, trometamol), or PEG-derived laxatives]?
3. Did you previously suffer from severe allergic reactions^*^ to infused or injectable drugs? (^*^history of anaphylactic shock that required the prescription of adrenaline, a life-threatening allergic reaction)?
4. Did you previously suffer from a severe allergic reaction of other origins^*^ (^*^oral medication, food allergy, latex, pollen, animals, dust mites, or insect venom)?

If the answer to question 1 was no, questions 2–4 were not asked. Persons with a yes answer to questions 2 and/or 3 were asked to get medical advice to assess their eligibility for the vaccination, either by contacting their general practitioner or by filling an online form sent to our allergy division (see results section). Persons who responded yes to question 4 only were eligible for a vaccination with 30-min surveillance. Thus, the high-risk profile was defined by a known allergy to one of the vaccine excipients (including polysorbate-80) or a history of a severe allergic reaction to an injectable drug containing, or possibly containing, one of the vaccine excipients. The standardized workup consisted of skin tests with one of the two anti-SARS-CoV-2 mRNA vaccines. Skin prick tests (SPT) with histamine and NaCl 0.9% were used as positive and negative controls, respectively. If skin tests returned negative (SPT 1:1, intra-dermal-reaction (IDR) 1:100), patients were offered a two-step (10–90%) vaccination protocol on the same day, with 30-min of surveillance after each injection. If skin tests returned positive, a complementary workup was done, including whenever possible both BNT162b2 mRNA and mRNA-1273 vaccines (SPT 1:1, IDR 1:100), polysorbate-80 1% (Hanseler AG, Herisau, Switzerland, SPT 1:1, IDR 1:10), PEG-2000 1% (Roth AG, Bern, Switzerland, dilution SPT 1:1, IDR 1:100), and trometamol 1% (Merk, Kenilworth, NJ, SPT 1:1 and IDR 1:100), the latter being an excipient of the mRNA-1273 vaccine. IDR with NaCl 0.9% was used as a negative control. Skin tests were performed on the anterior face of the forearm. SPT was performed with plastic devices (Stallerpoint, STALLERGENES GmbH, Kamp-Lintfort, Germany). IDR was performed with tuberculin syringes for the vaccines (1 ml syringes with 25 or 27G needles). The volume injected for IDR was adjusted to reach an initial wheal of 3–5 mm. All skin test solutions were prepared by the pharmacology center of the University Hospital of Lausanne. According to the European Network of Drug Allergy (ENDA) guidelines, a skin test was considered positive in the case of a papule of 3 mm or more, in comparison to the steady-state with erythema at 20 min. In case of a papule evolving from the steady-state but of < 3 mm, or if there was erythema absent in the negative control test (NaCl 0.9%), the test was considered inconclusive (suspicious).

### Post-vaccination Cohort

This cohort included all patients with suspected allergic reactions after the first or second injection with mRNA vaccine against COVID-19. Individuals with a suspected allergic reaction but who did not meet the anaphylaxis criteria as per the European Academy of Allergy and Clinical Immunology (EAACI) definition (11–13) received an appointment in our division of allergy for a standardized workup, consisting of skin testing with one of the two mRNA vaccines and, if negative, this was followed by vaccination the same day. The same complementary skin tests described above were performed if skin tests returned positive. After the first mRNA vaccine dose, individuals experiencing an anaphylactic reaction (as per EAACI definition) received an appointment for skin testing only (including one of the two mRNA vaccines, polysorbate-80 PEG-2000, and trometamol). Upon positive or inconclusive skin test results, we recommended a BAT to confirm the skin test results. In vaccine-hesitant patients with negative skin test results, we consider BAT testing to confirm skin test results for patient reassurance and increase vaccine adherence.

### Basophil Activation Tests

Peripheral blood was obtained from individuals from the pre- or post-vaccination cohorts. Briefly, 100 μl of peripheral blood per test condition was incubated with or without IL-3 (1 ng/ml) with different concentrations of vaccines (1, 0.1, and 0.01%) or polysorbate-80 (10 μg/ml, 2 μg/ml, and 0.4 μg/ml) for 30 min at 37°C, along with anti-CCR3 PE and anti-CD63 FITC, anti-CD203 APC antibodies (Biolegend, San Diego, CA). Control conditions included a medium-only negative control, a positive control, involving the crosslinking of the high-affinity Fc epsilon receptor (anti-IgE) (Beckmann-Coulter, Brea, CA), and a positive control independent of FcERI signaling, *N*-formylmethionyl-leucyl-phenylalanine (fMLP) (Sigma–Aldrich, St. Louis, Missouri). Flow cytometry data were collected on NovoCyte (Agilent Technologies, Santa Clara, CA). BATs were considered positive if two separate concentrations elicited a stimulation index of 2 or more for a given marker (CD63 or CD203c).

### Statistics

Multiple unpaired *t*-tests were performed using GraphPad Prism version 9 for Macintosh (GraphPad Software, San Diego, California USA). Mean and SD are shown. A normal distribution of the data was assumed. Fisher's exact tests were performed using IBM SPSS Statistics for Macintosh, Version 27.0.

## Results

### Pre-vaccination Cohort

**Figure 1:**
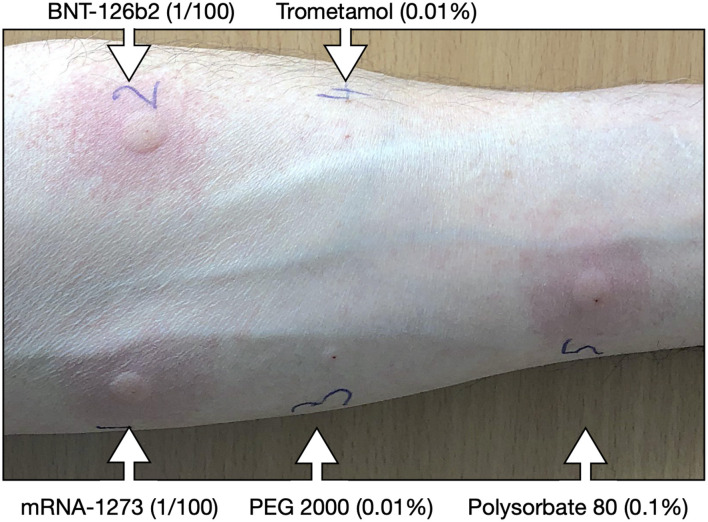
Skintesting with COVID-19 mRNA vaccines. Representative intradermal (IDR) skin tests results of a patient with a history of laxative anaphylaxis. The picture was taken 20 min after IDR for the BNT162b2 mRNA and mRNA-1273 vaccines (1/100), PEG 2000 1% (1/100), trometamol 1% (1/100). All dilutions were performed in NaCl 0.9%. PEG, polyethylene glycol; TRIS, trometamol; IDR, intradermal reaction.

**Figure 2:**
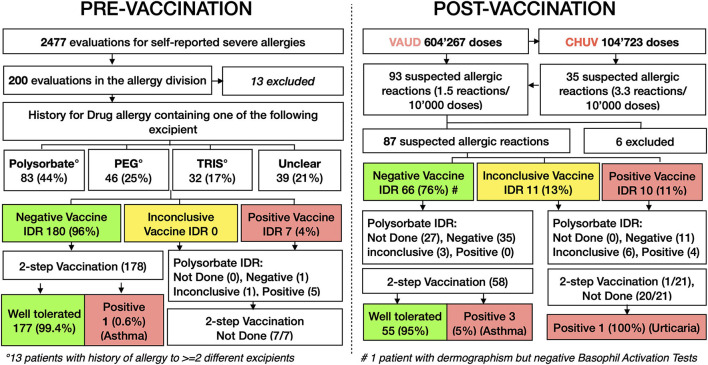
Flow chart pre- and post-vaccination. Flow chart of individuals from the pre-vaccination and post-vaccination cohorts, which were evaluated in the division of allergy at the university hospital of Lausanne. PEG, polyethylene glycol; TRIS, trometamol; IDR, intradermal reaction.

During the first 6 months of the vaccination campaign, we evaluated 2,477 online forms from vaccine candidates with allergies. We called in 200 persons with a high-risk profile for vaccine anaphylaxis for workup at our outpatient clinic. A total of 13 patients did not provide informed consent for this study and were therefore excluded from the analysis. The Mean age was 62.7-year-old, 145/187 (78%) were female, and 150/183 (82%) had a prior history of anaphylaxis (Table 1). One of the first cases with positive skin tests was a patient with a previous history of generalized pruritus, urticaria, dyspnea, and dysphonia minutes after starting preparation of macrogol 3,350 for a colonoscopy in 2020. The patient underwent SPT and IDR skin testing. SPT was inconclusive for the mRNA-1273 vaccine but otherwise negative. IDR was strongly positive for the BNT162b2 mRNA and mRNA-1273 vaccines and polysorbate-80 and negative for PEG-2000 and trometamol (Figure 1). We found six other patients with positive skin tests (IDR) for mRNA vaccines. All seven patients had positive IDR tests for both mRNA vaccines. Interestingly, 5/7 also had positive IDR for polysorbate-80, but none of them was positive for PEG-2000 or trometamol. In addition, none of the patients with an undefined history of anaphylaxis had positive skin tests with the vaccines (Figure 2, Table 2). Patients with negative skin tests for mRNA vaccine were offered a 2-step (10–90%) vaccination protocol, which 178/180 patients accepted. Vaccine tolerance was 99.4% (177/178 patients). One patient developed an isolated asthma attack, received adrenaline, and required 12-h monitoring at the emergency department.

**Table 1:** Characteristicsof patients included in the pre- and post-vaccination cohort.

	**Pre-vaccination Cohort**	**Post-vaccination Cohort**	***p*-value**
Total of patients	187	87	
Age (mean, +/–SD)	62.7 (+/−16)	48.4 (+/−15.8)	<0.01
Female	145 (78%)	78 (90%)	0.03
Negative skin Tests (SPT and IDR)*	180 (96%)	65 (76%)	<0.01
Positive SPT for either mRNA vaccines*	0	0	
Positive BNT-162b2 Skin IDR*	7 (4%)	7 (8%)	
Inconclusive BNT-162b2 IDR*	0	10 (12%)	
Positive mRNA-1273 Skin IDR*	7 (4%)	10 (12%)	
Inconclusive mRNA-1273 Skin IDR*	0	10 (12%)	
Prior history of allergy sharing additives with vaccines#	119 (64%)	3 (3%)	<0.01
Prior history of anaphylaxis (EAACI definition)	150/183* (82%)	34 (39%)	<0.01

# In 4 patients the history of anaphylaxis was not defined. *One patient had dermographism but negative basophil activation tests. For continuous variables, t-tests were performed. Fisher's exact tests were performed for non-continuous variables. IDR, intradermal reaction; SPT, skin prick test.

**Table 2:** Characteristics of patients with positive and negative skin tests in the pre-vaccination cohort.

	**Skin IDR Neg**	Skin IDR Pos^*^
	***n*= 180 (% or SD)**	***n*= 7 (% or SD)**
Age (mean)	62.6 (+/−16.3)	62.7 (+/−11.7)
Female	140 (78%)	6 (85%)
Prior anaphylaxis	145/175 (83%)	6 (85%)
Skin tests performed with mRNA-1273	95/180 (53%)	7 (100%)
Skin tests performed with BNT162b2	85 (47%)	7 (100%)
**History of allergy with drugs containing:**		
Polyethylene glycol	43 (24%)	3 (43%)
Polysorbate	81 (45%)	2 (29%)
Trométamol	30 (17%)	2 (29%)
Not defined	39 (22%)	0

*IDR, intradermal reaction*.

* *Including inconclusive intradermal skin tests*.

### Post-vaccination Cohort

In parallel, our division of allergy was in charge of evaluating suspected allergic reactions after the first or second dose. Suspected allergic reactions were defined as type I hypersensitivity symptoms (e.g. flush, dizziness, urticaria, angioedema, wheezing, shortness of breath, anaphylaxis) independently of their timing (Table 3). Thus, since the half-life of the mRNA vaccine is poorly defined, we intentionally tested individuals with delayed (>60 min) type I hypersensitivity symptoms. As of 16 June 2021, 604,267 doses =first (382,096) and second doses (222,107) of mRNA-based vaccine were injected in the canton Vaud. 241,103 (40%) were the BNT162b2 mRNA vaccine and 363,164 (60%) were the mRNA-1273 vaccine. As of 16 June 2021, 104,723 vaccine doses [=first (58,523) and second doses (46,200)] were administered at the University Hospital of Lausanne (CHUV). A total 94,017 (90%) were the BNT162b2 mRNA vaccine, and 10,706 (10%) the mRNA-1273 vaccine. By 16 June 2021, 35 suspected allergic reactions (3.3 reactions/10,000 doses) were reported at the vaccine center of CHUV, and overall, 93 from all vaccine centers in the canton Vaud (1.5 reactions/10,000 doses) (Figure 2).

**Table 3:** Characteristics ofthe 86 patients tested for the mRNA vaccines after experiencing a suspected allergy reaction immediately after immunization.

	**Skin IDR Neg**	Skin IDR Pos^*^	***p*-value**
Total of patients	65	21	
Age (mean +/–SD)	51.2(+/−15.1)	40.8 (+/−15.2)	<0.01
Female	57 (88%)	20 (95%)	0.33
BNT-162b2 (%)	43 (66%)	3 (14%)	<0.01
Prior allergy sharing additives with vaccines	2 (3%)	1 (5%)	0.71
Prior anaphylaxis	27 (41.5%)	7 (33%)	0.5
Prior allergy (any)	60 (92%)	12 (57%)	<0.01
Normal Basal Tryptase	50/51 (98%)	19/19 (100%)	0.5
**Symptoms**			
Cutaneous	53 (82%)	17(81%)	0.95
Respiratory	22 (33%)	6 (29%)	0.65
Digestive	7 (11%)	1 (5%)	0.41
Cardiovascular	2 (3%)	1 (5%)	0.71
**Treatment**			0.61
AntiH1, Steroids only	50 (77%)	17 (81%)	
Autoresolutive	12 (19%)	4 (19%)	
Epinephrine	3 (5%)	0	
**Timing**			0.36
Timing ≤ 30 min	42 (65%)	11 (52%)	
Timing 30–60min	2 (3%)	0	
Timing >60min	21 (32%)	10 (48%)	
Anaphylaxis EAACI criteria	16 (25%)	2 (10%)	0.14
**Brighton Scale**			0.3
I	5 (8%)	2 (10%)	
II	9 (14%)	0	
III	1 (2%)	0	
No criteria	50 (77%)	19 (91%)	
**Ring and Messmer Severity Scale**			0.24
I	40 (62%)	13 (62%)	
II	18 (28%)	8 (38%)	
III	7 (11%)	0	
IV	0	0	

*Fisher's exact tests were performed. For continuous variables, t-tests were performed. IDR, intradermal reaction*.

* *Including inconclusive intradermal skin tests*.

Among the 93 evaluated patients, 87 provided informed consent for the study. Five were suspected allergic reactions after the second dose. The mean age was 48.4 years, and 90% were women. In 36% of cases, the reactions occurred after 60 min. Three of 87 (3%) had a history of allergy to drugs sharing excipients with the mRNA vaccines (Table 1). Significantly fewer individuals had negative skin tests (SPT and IDR) compared to the pre-vaccination cohort (76% vs. 96%, respectively, *p* < 0.01, Table 1). Two patients refused the vaccine challenge. The interval between the first vaccination and the testing/revaccination was kept as close as possible to 4 weeks (mean 41 days, SD ± 14.95 days). Thus, in the majority of the cases, skin testing (if negative) and vaccination were set up the same day. Notably, over 95% (55/58) of individuals with negative skin tests (SPT and IDR) tolerated a two-step rechallenge protocol (Figure 2). Three individuals developed isolated asthma attacks, one after the 10% step and two after the 90% step. Two received adrenaline and required monitoring (<24 h) at the emergency department, one patient improved rapidly with a bronchodilator and anti-histamine only.

Workup yielded positive IDRs to the vaccine in 10/86 (11%). A total of 10/10 had positive IDRs for mRNA-1273, 7/8 had positive IDRs for BNT162b2, and 1/8 had an inconclusive IDR for BNT162b2. IDRs remained inconclusive in 11/86 (12%) patients. Importantly, SPT for either mRNA vaccines (undiluted) were negatives. Skin tests with PEG-2000 were negative in 19/21 cases and inconclusive in 2/2 (SPT inconclusive in 1 individual, otherwise negative). Skin tests (SPT and IDR) with trometamol were negative in all 21 patients. Finally, among individuals with inconclusive/positive IDR for mRNA vaccines, (10/21) 48% had inconclusive/positive IDR for polysorbate-80 (only 1 patient had positive SPT). Positive skin tests were significantly associated with younger age (p < 0.01) and the mRNA-1273 vaccine (*p* < 0.01) (Table 3). However, neither the symptoms, timing of symptoms, criteria for anaphylaxis, Brighton Score (level 0–3 defining the certainty of anaphylaxis), nor the Ring and Messmer Severity Scale (1–4 grading system of clinical anaphylaxis severity) were significantly more associated with skin test positivity to the mRNA vaccine (Table 3).

### Positive Vaccine Skin Tests Correlate With BAT Results

**Figure 3:**
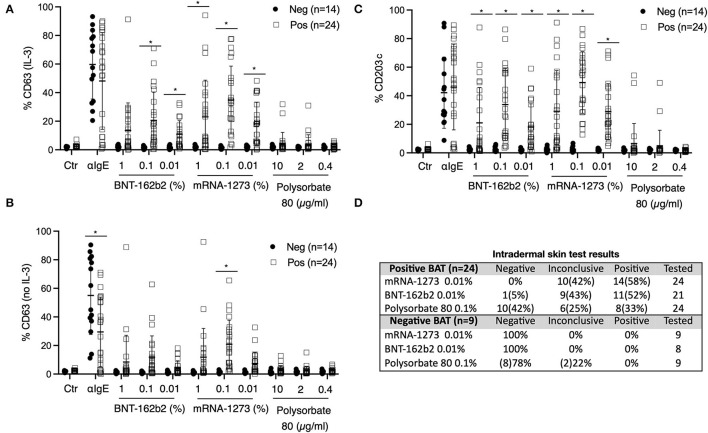
Basophil activation tests (BAT). **(A–C)** Percentage of CD63 and CD203c expression after gating on CCR3+ basophils. A total 27 of BATs were interpreted as positive and 17 as negative for sensitization to COVID-19 mRNA vaccines. **(A)** Peripheral blood was incubated with IL-3 and different concentrations of COVID-19 mRNA vaccines or polysorbate-80. Anti-IgE antibodies were used as positive control and medium-only as a negative control. The percentage of CD63 expression is shown. **(B)** Peripheral blood was incubated with different concentrations of COVID-19 mRNA vaccines or polysorbate-80 without IL-3. CD63 expression is shown. **(C)** Peripheral blood was incubated with different concentrations of COVID-19 mRNA vaccines or polysorbate-80. CD203c expression is shown. **(A–C)** Anti-IgE antibodies were used as positive control and medium-only as a negative control. **(D)** Correlation of BAT results with skin test results (when available). Multiple unpaired *T*-test was performed. Mean and standard deviation are shown. *p < 0.05. Neg, negative; Pos, positive.

To further investigate the inconclusive and/or positive IDR with either mRNA vaccine, we performed BAT in 24 individuals from the pre- and post-vaccination cohorts (Figures 3A–C). As controls, we included 14 individuals, among which four were healthy and vaccinated individuals without a history of allergy (skin tests not available), one had a suspected allergic reaction but dermographism (skin tests were not interpretable, the vaccine challenge was tolerated), two had inconclusive IDR for polysorbate-80 but were negative for the vaccine, and seven were vaccine-hesitant patients with a suspected allergic reaction but negative skin tests (SPT and IDR).

BATs were performed at the ADR-AC Laboratory in Bern, Switzerland. The interpretation was made independently of the skin test results. Strikingly, BAT results were consistently positive for inconclusive and/or positive skin tests with the vaccine, while individuals with negative skin test results had negative BAT results (Figure 3D). The results also showed cross-reactivity between the mRNA-1273 and BNT162b2 mRNA vaccines, which we also observed with skin testing. CD203c appeared to be the best marker to discriminate between positive and negative BAT results across all tested concentrations of vaccine (Figures 3A–C). Finally, while no significant difference was found for polysorbate-80 positivity between vaccine positive and negative BAT, we only observed a few outliers in vaccine-sensitized individuals.

### Vaccine-Induced Basophil Activation Is IgE-Dependent

**Figure 4:**
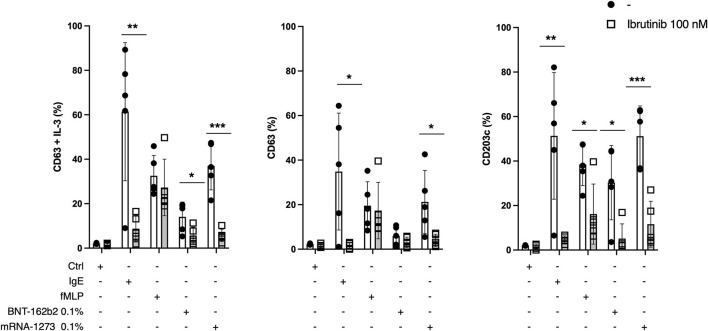
Ibrutinib and basophil activation tests (BAT). Percentage of CD63 (± IL3) and CD203c expression after gating on CCR3+ basophils in five individuals which were sensitized to the COVID-19 mRNA vaccines. A total of 100 nM of Ibrutinib was added to the control condition (medium-only), basophils were activated with anti-IgE monoclonal antibodies, or fMLP, or 0.1% of the BNT162b2 mRNA or mRNA-1273 vaccines. Multiple unpaired *T*-test was performed. Mean and SD are shown. *p < 0.05, **p < 0.01, ***p < 0.001.

Ibrutinib is a Bruton's tyrosine kinase inhibitor that targets IgE-dependent secretion of histamine in basophils (14). To investigate whether vaccine-induced upregulation of CD63 and CD203c is IgE-dependent, we performed a BAT in five vaccine-sensitized individuals with and without ibrutinib (Figure 4). As previously demonstrated (14), ibrutinib selectively inhibited CD63 upregulation upon basophil stimulation with anti-Fcε receptor I antibodies, but not with the bacterial peptide fMLP. Yet, we also observed a partial but significant inhibition of CD203c upon fMLP stimulation with ibrutinib, suggesting that this drug also has some IgE-independent activity. Importantly, in vaccine-stimulated conditions, we found a significant downregulation of basophils' CD63 and CD203c expression with ibrutinib, suggesting that the upregulation of those markers is, at least partially, IgE-dependent.

## Discussion

This is, to our knowledge, the largest study directly investigating vaccine sensitization in two cohorts of patients, either before vaccination in individuals with a high risk for anaphylaxis, or after vaccination in individuals with suspected allergic reactions. We showed that negative skin testing predicts vaccine tolerance independently of the severity of the initial reaction. These data emphasize the usefulness of IDR testing with the vaccine to decide whether patients are eligible for the primary vaccination or a booster dose. Importantly we have demonstrated that CD63 upregulation in basophils was at least partially IgE-dependent.

The incidence and/or prevalence of suspected allergic reactions for mRNA-based vaccines is intensely debated. Multiple definitions of anaphylaxis, data collected from national agencies/registries vs. prospective clinical studies, but also varying efforts made to determine the validity and causality of all suspected allergic reactions hampered these estimations (7, 15). Based on our experience, we estimated overall 1.5–3.3 suspected allergic reactions per 10.000 doses. These numbers however need to be taken with caution since 95% of them occurred after the first dose and we cannot exclude a selection bias for early investigations of patients waiting for their booster. Yet, considering the estimation of the Mass General Brigham hospital which found a rate of severe allergic reaction or anaphylaxis of 2.47 per 10,000 vaccinations, our results remain in the range of the existing literature (16). More information will come with ongoing studies prospectively evaluating the proportions of systemic allergic reactions to the BNT162b2 mRNA and mRNA-1273 vaccines (NCT04761822).

In this study, a third of the reactions started >60 min after vaccination. Delayed hypersensitivity type I symptoms have been reported after anti-SARS-CoV-2 vaccination (17–19). Yet, increasing evidence is now suggesting that delayed hypersensitivity is not a risk factor for a new allergic reaction upon vaccination. Xu et al. showed in a retrospective study that delayed allergic reactions, including hives and angioedema, did not preclude tolerance of subsequent booster doses in eight patients. Thus, many recommend not performing any tests for >4 h delayed reaction and vaccinating those individuals with 15–30 min of surveillance (20).

PEG is one important suspected culprit for mRNA-vaccine allergic reactions (1, 7, 9) 1, 7, 9 as it is known to provoke IgE-mediated anaphylaxis (4, 10, 21). Yet, the role of PEG testing for individuals with suspected allergic reactions to the first dose of mRNA COVID vaccine is currently being intensively studied and debated. A multidisciplinary group of international experts performed a systematic review and identified 21 studies on skin testing with PEG and/or polysorbate-80 (4, 7, 10, 22–29). They concluded with a general sensitivity of 58.8% and specificity of 99.5% (7). Wolfson et al., recently concluded on a limited role of PEG skin testing as individuals with suspected allergic reactions to the first dose of mRNA COVID vaccine could receive the second dose regardless of the skin test results (20). In our two cohorts of patients, PEG-2000 skin testing (SPT 1% and IDR 0.01%) was negative in the majority of cases and inconclusive in very few, which also speaks against the use of PEG testing to diagnose mRNA vaccine hypersensitivity.

Evidence shows that females make up a high percentage of those with suspected allergic reactions in our post-vaccination cohort. Yet, this finding has also been reported by others. In their observational study, the Mass General Brigham hospital showed that anaphylaxis cases after mRNA covid-19 vaccination were female in 94% of the cases (16). A total of 19/21 cases of anaphylaxis reported to the Vaccine Adverse Events reporting were also female (2). The recent position paper from the ENDA/EAACI also reviewed this and confirmed the very strong female predominance (30). The reasons for this observation remain unclear. It is tempting to hypothesize that females are more exposed to petroleum-based compounds with PEGs such as cosmetics, moisture-carriers, and thickeners and therefore more susceptible to developing anti-PEG antibodies.

This study has, however, important limitations in this regard. First, we intentionally used a low concentration of PEG-2000 to reduce the risk of anaphylaxis (10). In addition, it was recently recommended to perform only SPT and not IDR using a stepwise approach with PEG of different molecular weights (22). Secondly, we decided not to use surrogate drugs such as methylprednisolone acetate (PEG 3350) as recommended by others (4). This decision was made because of the many patients to be examined in a restricted time with limited resources. Finally, PEG-2000 testing has so far never been validated for diagnosing mRNA vaccine hypersensitivity (8, 31). Altogether, our results on PEG skin testing should be interpreted with caution.

In 4 cases out of 236 vaccinations, we observed isolated asthmatic reactions despite negative skin tests. Further investigations are needed to define the mechanisms involved in these reactions but non-IgE mechanisms have also been evoked to explain pseudo-allergic reactions to the newly developed mRNA-based vaccines. Those could be mediated in a complement-dependent manner as anti-PEG-IgM has been shown to trigger anaphylaxis in large animal models (32, 33). This was further suggested by Nadeau and colleagues who tested 11 individuals with anaphylaxis and could find IgG but not IgE against PEG (32). Yet, serological assays detecting anti-PEG IgE remain poorly standardized, ranging between 0.2 and 72% among healthy individuals (32, 34). Additionally, our results showed a strong correlation between IDR (not SPT) and BAT with significantly more sensitized individuals in the post-vaccination compared to the pre-vaccination cohort. Finally, BAT inhibition with ibrutinib also suggested that the upregulation of CD63 and CD203c in basophils is IgE-dependent. Thus, the results with ibrutinib are in line with those reported by the group Torres, who used wortmannin to selectively inhibit IgE-mediated CD63 upregulation (35).

The main limitation of this study is the lack of provocation tests in the vaccine-sensitized group, except for one patient who developed acute urticaria. Thus, we have not validated the specificity of the skin testing nor the BAT with a formal drug challenge. Yet, considering that drug allergies are routinely diagnosed in clinical practice with skin testing and BATs, we were not comfortable with challenging vaccine-sensitized patients. More data are to come with ongoing clinical trials evaluating the safety of administering a second dose of COVID-19 mRNA vaccines in individuals who experienced systemic allergic reactions to the first vaccine injection (NCT04977479). In our opinion, the most significant contribution of our study is to demonstrate that skin testing and BAT with mRNA vaccines are useful approaches to identifying vaccine sensitized patients. So far, recommendations have been scarce. Thus, the latest position paper from the ENDA/EAACI recommends only skin prick and not intradermal tests to avoid vaccine waste (30). Our results showed that STPs were limited as they turned in the majority negative, while BAT and IDR could identify vaccine sensitized patients. Thus, we found that IDR tests with mRNA-vaccines have a sensitivity of >98%. Such testing could help to evaluate and identify IgE-dependent anaphylaxis. Altogether, in our opinion, these results suggest that SPT may be omitted in the context of this pandemic to avoid vaccine waste. Instead, we would recommend performing IDR (1:100 dilution), which actually requires a smaller amount of vaccine than SPT.

We unexpectedly observed a significantly increased number of vaccine-sensitized patients in individuals who received the mRNA-1273 vaccine, although this vaccine is not associated with a higher prevalence of allergic reaction as compared to the BNT162b2 mRNA vaccine. Importantly, these results were confirmed by BAT and were specific to the post-vaccination cohort since 53% of individuals with negative skin tests in the pre-vaccination cohort were screened with the mRNA-1273 vaccine and not with the BNT162b2 mRNA. In our opinion, urgent studies are warranted to understand better the mechanisms and potential clinical consequences of vaccine-induced sensitization as they could be related to the dosage of mRNA (100 μg for mRNA-1273 vs. 30 μg for BNT162b2).

**Figure 5:**
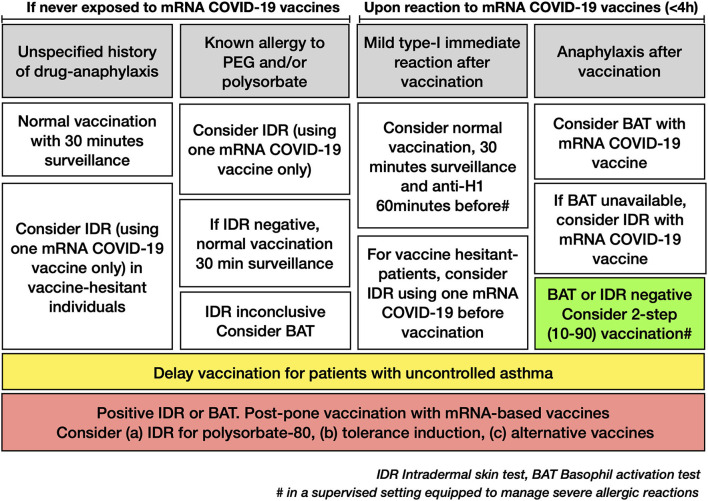
A suggested algorithm for the management of allergic patients with possible hypersensitivity against mRNA-based COVID-19 vaccines.

A recent systematic review and meta-analysis of case studies and case reports concluded on low risk for severe allergic reactions upon rechallenge of patients who experienced an immediate allergic reaction to their first dose (36). Considering these data, we propose that patients with immediate or delayed non-severe type-1 allergic reactions be directly challenged using a full vaccination protocol with 30 min surveillances and an anti-histamine as a premedication (Figure 5). Those challenges need yet to be performed in a supervised setting equipped to manage severe allergic reactions. For patients with anaphylaxis after injection of an mRNA-based vaccine, we still recommend performing a BAT and if not available, an IDR before vaccination to identify vaccine hypersensitivity. A two-step vaccination protocol could be considered for those individuals with negative tests again in a supervised setting. Importantly, IDR may represent an essential tool for vaccine-hesitant patients to receive the booster associated with a substantial gain in antibody neutralizing capacities and immune protection (37, 38). Thus, allergic symptoms after vaccination have been shown to contribute to incomplete vaccination (39).

Based on the results of this study, it seems also reasonable to restrict mRNA vaccine skin tests before vaccination to patients with documented or highly suspected allergy to PEG and/or polysorbate and to consider them only in vaccine-hesitant individuals with an unspecific history of drug-anaphylaxis or multiple drug allergies to increase global coverage of vaccination (Figure 5).

In conclusion, a two-step 10–90%-vaccination protocol was tolerated in 232/236 (98%) of individuals with negative intradermal tests, suggesting that split-dose vaccination can be safely administered upon negative testing. Yet, four reactions were not prevented with the proposed intradermal testing. While the mechanisms behind these reactions remain unclear, most patients had previous asthma-like reactions. Thus, we recommend postponing the vaccination of patients with uncontrolled asthma as a standard of care before any provocation tests. Further studies are warranted to define optimal management of vaccine-sensitized patients, in particular regarding tolerance induction protocols or the use of alternative vaccines.

## Data Availability Statement

The data that support the findings of this study are available on request from the corresponding author. The clinical data are not publicly available due to ethical restrictions.

## Ethics Statement

The study was approved by the local Ethics Committee of the canton of Vaud, Switzerland (BASEC number 2021-00735). The patients/participants provided their written informed consent to participate in this study. Written informed consent was obtained from the individual(s) for the publication of any potentially identifiable images or data included in this article.

## Author Contributions

YM: conceptualization, initiation of the study, and supervision. FS and YM: formal analysis and writing—original draft. DY, FS, TH, AM, CG, SM, and CR: contribution to the design of the study. FS, RM-A, LC, and VM-B: collection of the data. DY and KK: BAT analysis. TH and CR: writing—reviewing and editing. All authors contributed to the article and approved the submitted version.

## Funding

YM is supported by a grant of the Gabriella Giorgi-Cavaglieri Foundation. Open access funding provided by University of Lausanne.

## Conflict of Interest

DY and KK are employees of ADR-AC GmbH Switzerland. The remaining authors declare that the research was conducted in the absence of any commercial or financial relationships that could be construed as a potential conflict of interest.

## Publisher's Note

All claims expressed in this article are solely those of the authors and do not necessarily represent those of their affiliated organizations, or those of the publisher, the editors and the reviewers. Any product that may be evaluated in this article, or claim that may be made by its manufacturer, is not guaranteed or endorsed by the publisher.
