# Erratum: Intradermal testing with COVID-19 mRNA vaccines predicts tolerance

**DOI:** 10.3389/falgy.2023.1183702

**Published:** 2023-04-03

**Authors:** Frontiers Production Office

**Affiliations:** Frontiers Media SA, Lausanne, Switzerland

**Keywords:** SARS-CoV-2, allergy, vaccine, polyethylene glycol, PEG, COVID-19, anaphylaxis, basophil activation test

An Erratum on Intradermal testing with COVID-19 mRNA vaccines predicts tolerance By Stehlin F, Mahdi-Aljedani R, Canton L, Monzambani-Banderet V, Miauton A, Girard C, Kammermann K, Meylan S, Ribi C, Harr T, Yerly D and Muller YD. (2022) Front. Allergy 3:818049. doi: 10.3389/falgy.2022.818049

An omission to the **Funding** section of the original article was made in error. The following sentence has been added: “Open access funding provided by University of Lausanne”.

The original version of this article has been updated.

