# Supplementary figures and images for: Intradermal Testing With COVID-19 mRNA Vaccines Predicts Tolerance
Source: Front Allergy. 2022 May 31;3:818049. doi: 10.3389/falgy.2022.818049 (PMC9552867; doi:10.3389/falgy.2022.818049)

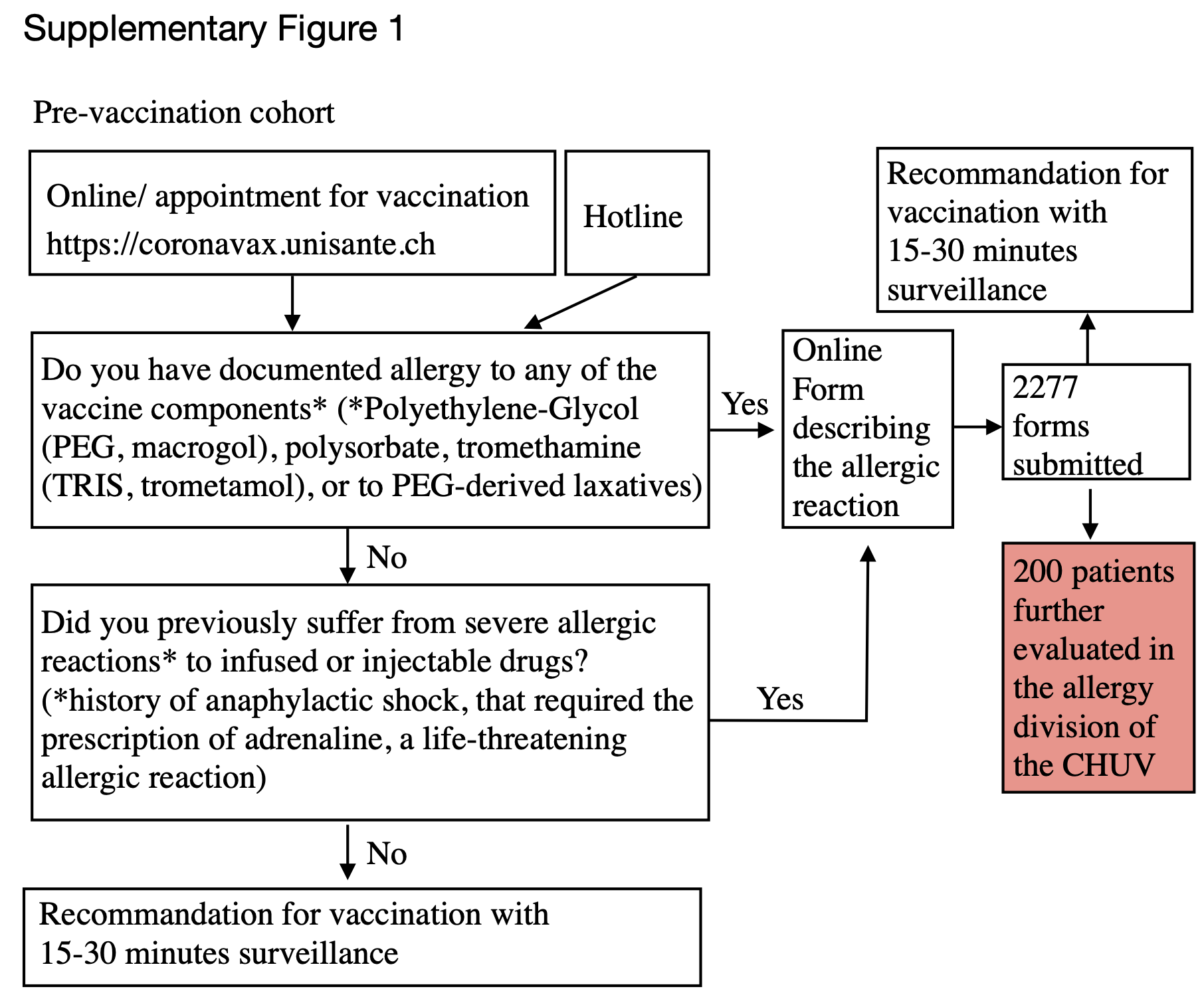

Supplement: Supplementary file 1 [file Image_1.TIFF]
